# Impact of health education on knowledge retention among caregivers of hypertensive patients: A prospective cross-sectional study in rural Malawi

**DOI:** 10.1371/journal.pone.0317684

**Published:** 2025-02-03

**Authors:** Chikondi Maluwa, Sitalire Kapira, Hataichanok Chuljerm, Wason Parklak, Kanokwan Kulprachakarn

**Affiliations:** 1 Research Institute for Health Sciences, School of Health Sciences Research, Chiang Mai University, Chiang Mai, Thailand; 2 Ministry of Health, Neno District Health Office, Neno, Malawi; 3 Malawi College of Health Sciences, Blantyre Campus, Blantyre, Malawi; 4 Partners in Health, Neno Office, Neno, Malawi; 5 Research Institute for Health Sciences, Research Center for Non-infectious Diseases and Environmental Health, Chiang Mai University, Chiang Mai, Thailand; George Washington University School of Medicine and Health Sciences, UNITED STATES OF AMERICA

## Abstract

Hypertension is a widespread and life-threatening condition affecting one-third of adults globally. In low- and middle-income countries, like Malawi, the burden of hypertension is escalating due to inadequate healthcare resources and lifestyle changes. Family members often become primary caregivers, playing a crucial role in managing hypertension through support and adherence to treatment. This study examined caregivers’ knowledge retention by evaluating their pre- and post-health education knowledge levels. This was a prospective cross-sectional study in Neno, Malawi, a rural setting. 422 caregivers were enrolled from the Integrated Chronic Care Clinic (IC3). A structured questionnaire was used to collect baseline, post-health education, and week six data. Using SPSS V 22.0, comparison of knowledge, attitude, and practices (KAP) scores, correlation between KAP and between KAP and social demographic characteristics were done using Wilcoxon signed-rank test, Pearson correlation, and independent t-test respectively. Among the 422 caregivers who participated in the study, 267 (63.2%) were females and mean age was 44.94 years. The baseline mean knowledge level score was 9.5 (38.0%) and rose to 21.08 (84.3%) p = 0.000 immediate post-health education and a 2.1% decrease 20.54 (82.2%) p<0.001 at week six from the immediate post health education score. Attitude improved from 16.76 (93.1%) at baseline to 17.74 (98.6%) at the six-week mark. Similarly, the mean practice score rose from 25.24 (78.9%) at baseline to 27.42 (85.7%) at week six. There was a positive correlation between KAP while age had a negative correlation with knowledge (r = -0.146; p = 0.003). There was a significant difference between different education levels on knowledge retention p = 0.009. There was a positive and good knowledge retention among caregivers of hypertensive patients after health education at the week six mark. With improved knowledge and the ability to retain it resulting in improved attitude and practices, caregivers are a cornerstone for continued and improved hypertension care for the patients.

## Introduction

Hypertension is a prevalent and life-threatening condition that affects 1 in 3 adults worldwide, significantly increasing the risk of stroke, heart attack, heart failure, kidney damage, and various other health complications. Between 1990 and 2019, the number of people living with hypertension doubled from 650 million to 1.3 billion, with nearly half remaining unaware of their condition [1]. Over three-quarters of adults with hypertension reside in low- and middle-income countries (LMICs), where non-communicable diseases (NCDs) are rapidly increasing [2].

In Malawi, 33% of the population suffers from hypertension, making it the leading cause of premature death related to NCDs [3]. Contributing factors include aging, smoking, excessive alcohol consumption, obesity, physical inactivity, and inadequate diets [4]. However, most hypertension cases go undetected and untreated due to limited healthcare resources [5].

In rural settings where healthcare services are often insufficient, family members frequently take on the role of caregivers for hypertensive patients. These caregivers, many of whom lack formal education, rely heavily on information provided by healthcare professionals [6]. However knowledge retention is a significant challenge among caregivers, directly influencing their ability to facilitate behavioral changes in patients [7]. Limited knowledge can hinder individuals from seeking medical attention, adhering to healthcare advice, and attending to follow-up appointments, potentially leading to complications [8]. Improving their knowledge through targeted health education and training enhances their attitude and practices, ultimately leading to better patient outcomes through improved quality of care they provide [9].

Health education plays a vital role in disease prevention and in mitigating the impact of NCDs by enhancing community awareness [10]. Studies have shown that health education can significantly improve patient outcomes. For example, Individual and Family Educative-Supportive Program among People with Heart Failure and methods such as teach-back, multimedia, and blended training have enhanced self-care behaviors and knowledge among caregivers of patients with heart failure while reducing the disease burden [11,12]. Similarly, planned health education has been effective in improving the knowledge and competence of caregivers providing home care for post-stroke patients [13]. However, there is a notable gap in the literature regarding the impact of health education on knowledge retention among caregivers of hypertensive patients in rural settings. Hypertension prevention and management in these areas faces unique challenges, including limited resources in diagnosing and treating the disease, lack of disease knowledge among the communities and delays in seeking medical attention [14]. Addressing these issues requires strategies that integrate individual, community, and healthcare service levels [15].

Thus, health education emerges as a cost-effective strategy to prevent hypertension and mitigate its burden. This prospective cross-sectional study aimed to evaluate the effectiveness of health education on knowledge retention among caregivers of hypertensive patients, focusing on its role in hypertension prevention and management in rural Malawi.

## Methodology

### Study design and setting

The researchers conducted a prospective cross-sectional study in Neno, Malawi a rural setting utilizing the Integrated Chronic Care Clinics (IC3) at Neno District Hospital and Lisungwi Community Hospital. Neno has a population of 146,236 and is considered one of the hardest-to-reach areas in the country. In 2018, there were more than 3000 patients enrolled in IC3 of which 60% were due to hypertension. 20% of the enrolled hypertensive patients defaulted from care [3].

### Study population

The participants were selected from the Integrated Chronic Care Clinic (IC3) at Neno District Hospital and Lisungwi Community Hospital. These were caregivers who came to the clinic with their patients. Participants eligible for the study were those who were 18 years and above and had been taking care of the patients for the past 6 months. Those with psychiatric disorders and who did not consent to their participation were excluded. Using the Charan and Biswas formula [16] (Z = 1.96 and alpha error = 0.05) and assuming 50% prevalence of KAP among caregivers in Neno, the minimum sample size required was 384. Participants meeting the inclusion criteria were identified at the IC3 clinic by the data entry clerk. The recruitment period was from November 27, 2023, to December 29, 2023.

### Research tool and data collection

Data were collected from November 2023 to February 2024 using a structured questionnaire that was developed based on literature review and expert opinions [17–24]. The questionnaire was approved by the Research Institute for Health Sciences questionnaire approval committee on July 3, 2023, with approval number [002/ 2023] before use. The structured questionnaire comprised of two parts (see S1 File):

Part 1: Demographic information of caregivers which includes age, gender, education level, relationship with the patient, location (village), occupation, religion and date of diagnosis.
Part 2: Knowledge, attitude, and practice of caregivers towards hypertension, its management and prevention. In the Knowledge section, answering options “Yes and No” were utilized. A total score of 25 points was allocated and participants received 1 point for every correct answer and 0 points for incorrect answers. For the Attitude section, a 3-point Likert scale was utilized, with response options including 1) "Disagree", 2) "Not sure", and 3) "Agree”. A total score of 18 points was allocated. Similarly, in the Practice section, a 4-point Likert scale was employed, with response options: 1) "Never", 2) "Rarely", 3) "Sometimes", and 4) "Always". A total score of 32 points was allocated.

The interviewer conducted health education following the baseline assessment. Health education included an overview of hypertension, signs, and symptoms, risk factors, prevention, and management. Subsequently, another assessment was administered to evaluate the caregivers’ acquired knowledge soon after the health education. This session took at least 30 to 40 minutes per participant. The assessment of knowledge retention was conducted six weeks after the immediate post-health education, and researchers employed the same structured questionnaire used in both the baseline and immediate post-health education assessments.

**Fig 1:**
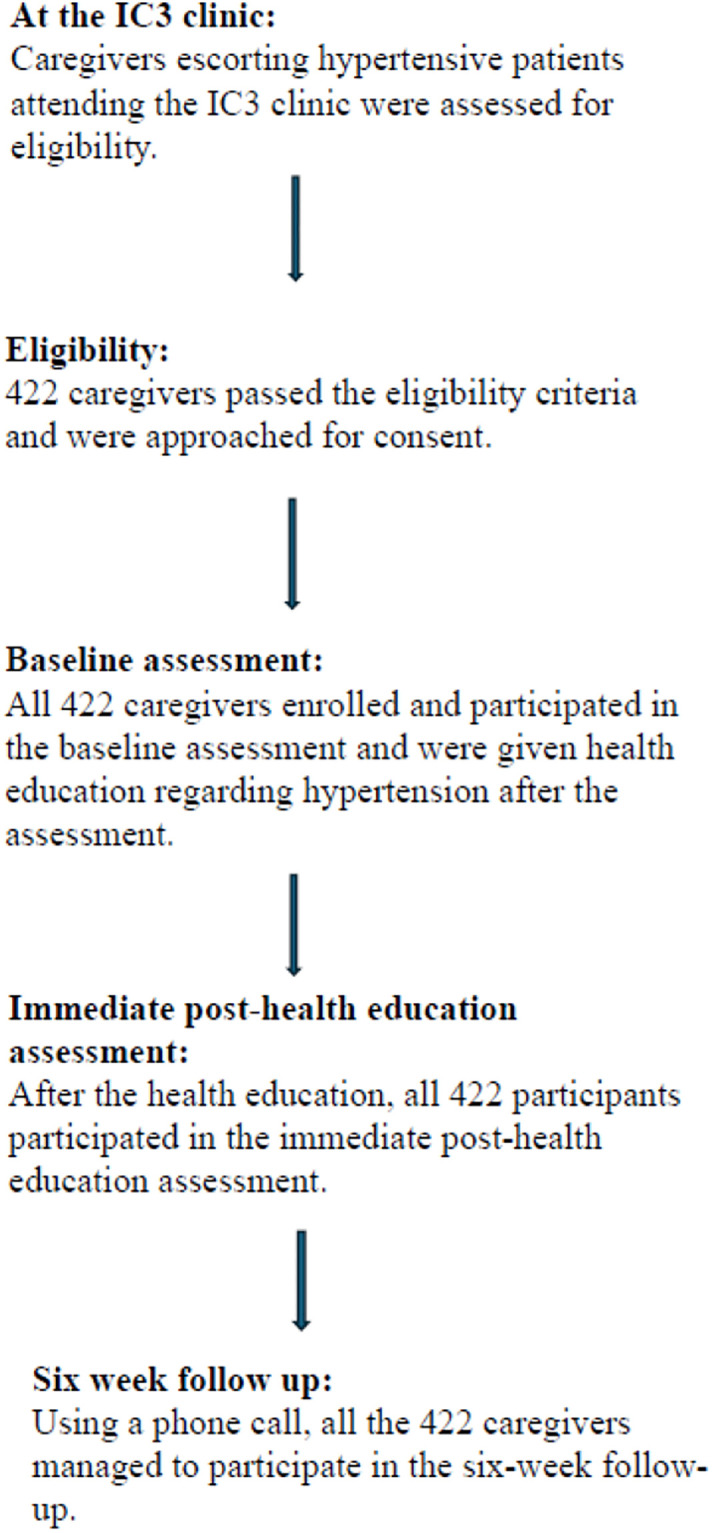
Participants enrollment.

The initial and post-health education assessments were done in person because the participants were already at the clinic. The Knowledge retention assessment was done using a phone interview to reduce the transportation cost of the participants as well as to avoid inconveniences because it was outside the clinic visiting date (Fig 1).

### Ethical committee approval

The study was approved by the Research Institute for Health Sciences (RIHES), Chiang Mai University Ethics Committee, Thailand [Approval No.60/2023], and Malawi National Health Sciences Research Committee [Protocol # 23/10/4216]. The researchers also got permission from the Neno District Health Office to conduct the study in Neno. Before data collection, participants were briefed on the study’s objectives and procedures, and written informed consent was obtained from all participants. The respondents were free to discontinue their involvement in the study at any time, as it was entirely voluntary. Additionally, they were free to decline to respond to any questions that made them uncomfortable. All their privacy was protected during the research.

### Data management and statistical analysis

Each section of the KAP assessment was graded based on allocated scores. Using modified Blooms cut off measures and cut-off points from other studies [17,19,21,24,25], the participants’ overall knowledge level and practices were categorized as good, moderate/fair, or poor if the total score was 80–100%, 60–79%, or less than 60%, respectively. Attitude was categorized as positive or negative if the total score was 60–100% or less than 60%, respectively.

The data extracted from Microsoft Excel was imported into Statistical Package for Social Science (SPSS) version 22.0. Descriptive statistics, including counts, percentages, and proportions for all categorical variables, were computed. To determine the total KAP scores for each respondent regarding hypertension, responses to each question were appropriately scored and summed for each section. The mean score was then used to assess the level of KAP related to hypertension and its contributing factors at baseline, post-health education, and during the week six assessments, as well as to evaluate knowledge retention. The Wilcoxon signed-rank test was used to compare the initial and the two post-health education scores. The correlation between KAP scores and between KAP and age was examined using a Pearson correlation test. Additionally, the variation in KAP scores among different sociodemographic groups was analyzed using an independent t-test.

## Results

### Caregivers’ socio-demographic characteristics

Among the 422 caregivers who participated in the study, 248 (58.8%) were from Lisungwi Community Hospital IC3 clinic, 267 (63.2%) were females and the average age was 44.94 ± 9.89 years. Regarding education level, 68.0% (n = 287) of the caregivers had a lower level of education. Over half of the caregivers (63.8%, n = 266) were non-blood-related (spouses), while 25.8% (n = 109) were blood-related (children). Most of the caregivers were unemployed (62.5%, n = 264). All participants were followers of the Christian faith, and a significant portion (27.4%, n = 116) had their patients in care for more than 5 years. Furthermore, almost all of them (97.8%, n = 413) obtained their knowledge from healthcare providers (Table 1).

**Table 1:** Socio-demographic characteristics of hypertensive patients’ caregivers (n = 422).

Characteristics	Males, n (%)	Females, n (%)	Total, n (%)
**Location**			
Neno Hospital	65 (15.4)	109 (25.8)	174 (41.2)
Lisungwi	90 (21.4)	158 (37.4)	248 (58.8)
**Age (years)**			
18–44	45 (13.1)	164 (38.8)	209 (51.9)
<44	100 (23.7)	103 (24.4)	203 (48.1)
**Level of education**			
Lower level of education	87 (20.6)	200 (47.4)	287 (68.0)
Higher level of education	68 (16.2)	67 (15.9)	135 (32.0)
**Relationship with patient**			
Blood related^c^	37 (8.8)	116 (27.5)	153 (36.3)
Non-blood related^d^	118 (28.0)	151 (35.6)	266 (63.8)
**Patient’s date of Diagnosis/Duration in care**			
<1–5 years	111 (26.4)	195 (46.2)	306 (72.6)
<5 years	44 (10.4)	72 (17.0)	116 (27.4)
**Occupation**			
Non-employed^e^	76 (18.1)	188 (44.5)	264 (62.5)
Employed^f^	79 (18.7)	79 (18.7)	158 (37.4)
**Religion**			
Christianity	155 (36.7)	267 (63.3)	422 (100)
Muslim	0 (0.0)	0 (0.0)	0 (0.0)
Others	0 (0.0)	0 (0.0)	0 (0.0)
**Source of knowledge**			
Health care provider	150 (35.5)	263 (62.3)	413 (97.8)
Internet	9 (2.1)	8 (1.9)	17 (4.0)
Support group	0 (0.0)	0 (0.0)	0 (0.0)
Community health worker	3 (0.7)	12 (2.9)	15 (3.6)
Others	2 (0.5)	1 (0.2)	3 (0.7)

Note:

^a^Primary and no education;

^b^Secondary and college education;

^c^Participants who were children and parents of the patients;

^d^Spouces and others;

^e^Farmers, students and housewives;

^f^Business people and those employed in different sectors.

### Level classification and summary of total scores of knowledge level, attitude, and practice

**Fig 2:**
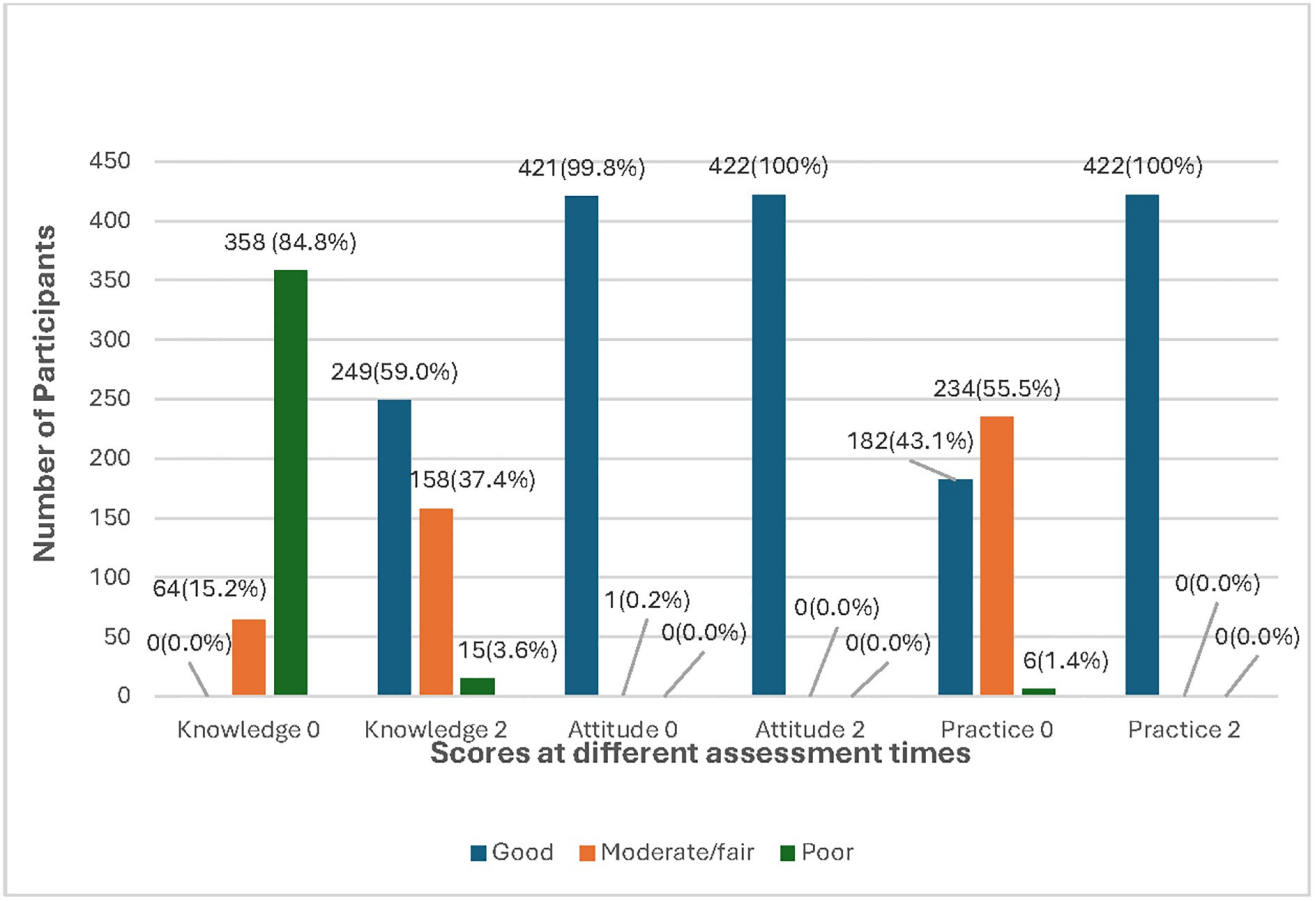
Participants’ summary of total KAP scores at different assessment times.

The knowledge level and practice assessment scores were categorized into three levels, good (80–100%), moderate/fair (60–79%), and poor (less than 60%) while attitude assessment scores were categorized into two levels, positive (60–100%) and negative (<60%). For knowledge level, participants scoring 20–25 points were considered to have good knowledge level, 15–19 points was moderate/fair, and 0–14 points was poor. The attitude score categories were 11–18 points as positive and 0–10 as negative. Practice scores were grouped into good (26–32 points), moderate/fair (19–25 points), and poor (0–18 points). The total score thresholds for each category are shown in Table 2, Fig 2.

**Table 2:** Level classification and summary of total scores of KAP towards hypertension among caregivers at baseline assessment and at week six assessment (n = 422).

% Score	Knowledge			Practice			Level	% Score	Attitude			Level
	Score	n	%	Score	n	%			Score	n	%	
**Baseline Assessment**												
**80–100**	20–25	0	0	26–32	182	43.1	**Good**	60–100	11–18	422	100	**Positive**
**60–79**	15–19	64	15.2	19–25	234	55.5	**Moderate/ Fair**					
**<60**	0–14	358	84.8	0–18	6	1.4	**Poor**	<60	0–10	0	0.0	**Negative**
**Week Six Assessment**												
**80–100**	20–25	249	59.0	26–32	422	100	**Good**	60–100	11–18	422	100	**Positive**
**60–79**	15–19	158	37.4	19–25	0	0.0	**Moderate/ Fair**					
**<60**	0–14	15	3.6	0–18	0	0.0	**Poor**	<60	0–10	0	0.0	**Negative**

### KAP assessment

**Fig 3:**
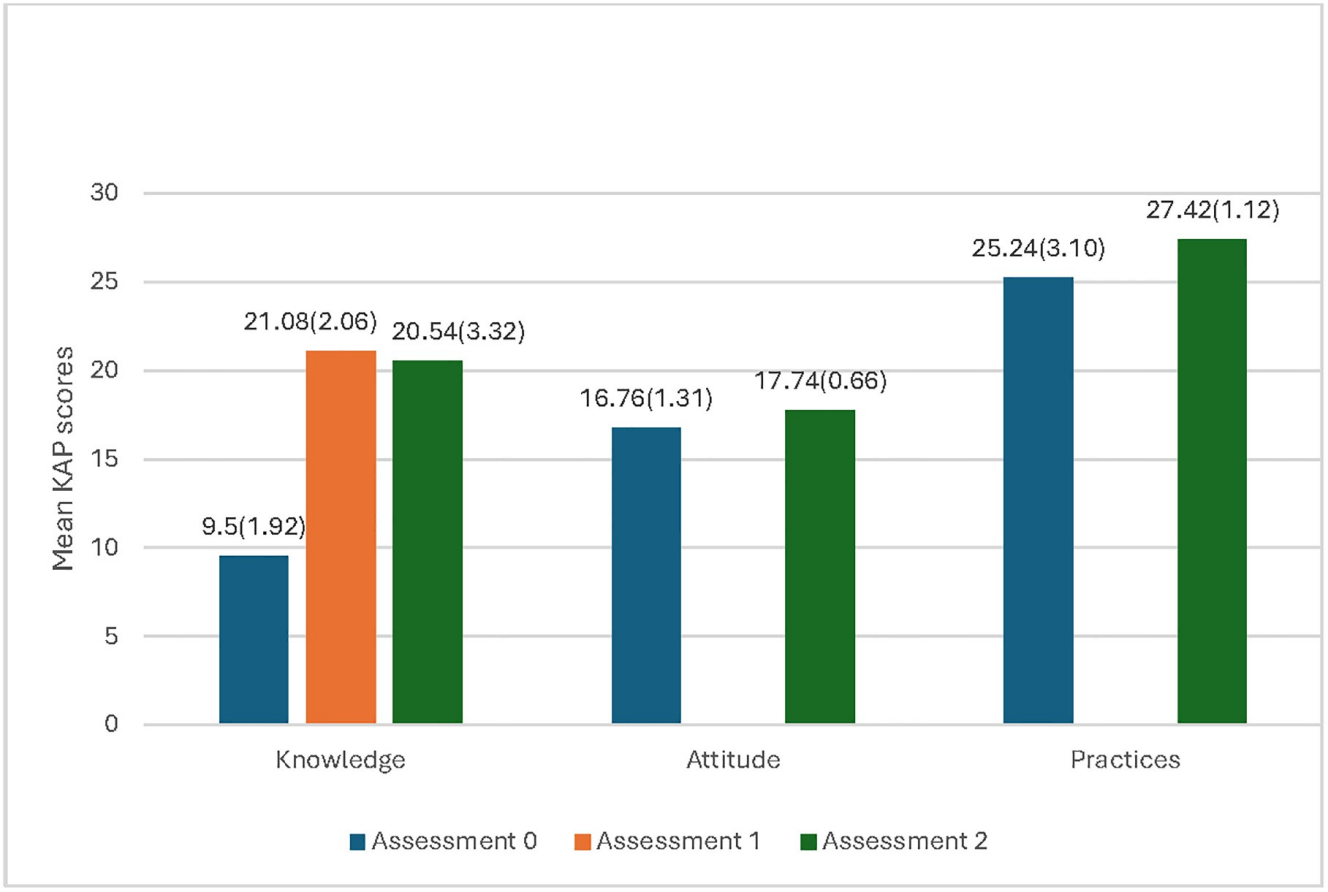
Mean KAP scores and standard deviation at different assessment times.

Following the health educational session, a significant increase in the mean Knowledge level, Attitude, and Practice (KAP) scores was observed among all participants compared to baseline measurements (Table 3, Fig 3). Participants demonstrated notable improvement in knowledge level, with scores rising from a baseline mean of 9.5 (38.0%) to 21.08 (84.3%, p <0.001) immediately post-health education. All 422 participants enrolled in the study successfully completed the six-week assessment period. However, at the end of six weeks, the mean knowledge level score showed a 2.1% decrease to 20.54 (82.2%, p <0.001) compared to the immediate post-health education score of 21.08 (84.3%). In terms of attitude, there was an increase in the mean score from the baseline of 16.76 (93.1%) to 17.74 (98.6%, p <0.001) at six weeks. Similarly, the mean practice score also increased from 25.24 (78.9%) at baseline to 27.42 (85.7%, p <0.001) at six weeks. It’s worth noting that no immediate post-health education assessment was conducted for attitude and practice.

**Table 3:** KAP assessment scores (n = 422).

	Baseline (Assessment 0)		Immediate post-health education (Assessment 1)			Week Six (Assessment 2)			
	*n*	Mean (%) (SD)	*n*	mean (%) (SD)	*p*-value*	*n*	mean (%) (SD)	*p*-value*	*p*-value^
Knowledge	422	9.5 (38.0) (1.92)	422	21.08 (84.3) (2.06)	<0.001	422	20.54 (82.2) (3.32)	<0.001	<0.001
Attitude	422	16.76 (93.1) (1.31)				422	17.74 (98.6) (0.66)	<0.001	
Practice	422	25.24 (78.9) (3.10)				422	27.42 (85.7) (1.12)	<0.001	

* Wilcoxon signed-rank test compared to baseline.

^ Wilcoxon signed-rank test compared to post-health education.

SD Standard deviation.

### Comparison of correlation between KAP and between age and KAP regarding hypertension at different assessment times

To compare the relationship between KAP scores and between age and KAP scores at baseline and those at six weeks post-health education, a bivariate analytic model was employed to ascertain the correlation (Table 4). Knowledge demonstrated a notably significant positive association with attitude (r = +0.255; p < 0.001) and practice (r = +0.252; p < 0.001) at baseline. Conversely, there was no statistically significant correlation between attitude and practice regarding hypertension at baseline (r = +0.064; p = 0.190). However, following post-health education at week six, a fair positive correlation emerged between knowledge and attitude, knowledge and practice, and attitude and practice (r = +0.381; p <0.001), (r = +0.396; p <0.001), and (r = +0.217; p <0.001), respectively. Age did not exhibit any significant correlation with either the knowledge score (r = +0.034; p = 0.490) or the practice score (r = +0.043; p = 0.382) at baseline assessment. However, there was a noteworthy and positive correlation between age and attitude score at baseline (r = +0.233; p<0.001). Conversely, following health education at the six-week mark, no correlation was found between age and attitude score (r = -0.061; p = 0.221) or age and practice score (r = -0.028; p = 0.562). Notably, there was a negative correlation observed between age and knowledge score at the six-week mark post-health education (r = -0.146; p = 0.003).

**Table 4:** Pearson correlation test, correlations between KAP and correlations between age and KAP at baseline and six weeks post-health education (n = 422).

Variables	Baseline			Six weeks		
	*r*-value	*p*-value	Interpretation	*r*-value	*p*-value	Interpretation
**Knowledge and Attitude**	+0.255	<0.001**	A fair, positive correlation	+0.381	<0.001**	A fair, positive correlation
**Knowledge and Practice**	+0.252	<0.001**	A fair, positive correlation	+0.396	<0.001**	A fair, positive correlation
**Attitude and Practice**	+0.064	0.190	No correlation	+0.217	<0.001**	A fair, positive correlation
**Age and Knowledge**	+0.034	0.490	No correlation	-0.146	0.003**	Negative correlation
**Age and Attitude**	+0.233	<0.001**	A fair, positive correlation	-0.061	0.221	No correlation
**Age and Practice**	+0.043	0.382	No correlation	-0.028	0.562	No correlation

** Correlation is significant at the 0.01 level (2-tailed).

### Comparing knowledge retention between different genders, educational levels, and occupations amongst caregivers of hypertensive patients

An independent t-test was employed to conduct a statistical comparison of knowledge retention for hypertension across different demographic groups. Specifically, we compared males (n = 155) and females (n = 267), individuals with higher levels of education (n = 135), which included secondary and college education, and those with lower levels of education (n = 287), encompassing individuals with no education and only primary education. Additionally, we compared participants based on their employment status: non-employed participants (n = 264), consisting of farmers, students, and housewives, and employed participants (n = 158), including businesspeople and individuals employed in various sectors. The results revealed a significant difference in knowledge retention among different education level groups (p = 0.009), whereas no significant differences were observed in attitude and practice. There were no significant differences observed in knowledge retention based on gender or occupation (Table 5).

**Table 5:** Comparison of knowledge retention among different socio-demographic factors using independent t-test (n = 422).

Groups being compared	n	Knowledge scores		Attitude scores		Practice scores	
		Mean (SD)	*p*-value	Mean (SD)	*p*-value	Mean (SD)	*p*-value
**Gender**							
Male	155	20.27 (3.29)	0.209	17.70 (0.70)	0.342	27.35 (1.09)	0.334
Female	267	20.69 (3.34)		17.77 (0.64)		27.46 (1.14)	
**Education Level**							
Lower level of education	287	20.83 (3.39)	0.009*	17.76 (0.64)	0.375	27.49 (1.14)	0.089
Higher level of education	135	19.93 (3.10)		17.70 (0.71)		27.29 (1.07)	
**Occupation**							
Non-employed	264	20.79 (3.23)	0.138	17.74 (0.67)	0.967	27.47 (1.12)	0.281
Employed	158	20.23 (3.46)		17.75 (0.66)		27.35 (1.12)	

*Significant at p < 0.05.

## Discussion

This prospective cross-sectional study findings show improvements from baseline in participants’ knowledge level (38.0%), immediately post-health education (84.3%) and at six weeks (82.2%), demonstrating the importance of health education and the ability to retain knowledge by the caregivers. Interestingly, even though participants in this study had varying educational backgrounds, changes in immediate post-health education knowledge were similar to those found in previous research [26–28].

Although health education is done almost daily at the clinic to the clients by the health care provider before they start rendering services, the baseline knowledge level score was still poor at 38.0%. After one-on-one health education sessions, there was an increase in the knowledge gained and knowledge retained. These findings highlight the need for tailored educational approaches to address different caregiver needs, emphasizing the importance of integrating caregiver education programs into routine healthcare services to strengthen hypertension prevention and management in resource-limited settings. With shortages in human resources in rural areas often coupled with the responsibility burden of clinicians [29], health education is a key to preventing and managing diseases. Lack of knowledge results in negative attitude and poor practices affecting the quality of care [30].

Gender disparities were also observed, with female caregivers having limited knowledge level but more positive attitudes towards hypertension management compared to their male counterparts at baseline. After the educational intervention, females scored higher in knowledge level, exhibited more positive attitudes, and demonstrated good practices related to patient care. This gender disparity was attributed to women’s primary caregiving role and more frequent interactions with healthcare providers [6].

The study also found that higher educational levels were associated with better knowledge level, positive attitudes, and good practices (KAP) regarding hypertension at baseline. However, at the six-week follow-up, lower educational level was associated with better KAP scores, possibly due to overconfidence in existing knowledge among those with higher education level [31]. This emphasizes the importance of education in empowering caregivers with the knowledge and skills necessary for effective health management. To address these challenges, there is a need for comprehensive educational programs. These programs should be designed to provide caregivers with the necessary knowledge and skills to effectively care for their loved ones, presented in a manner that is easily understandable to all participants, regardless of their educational background. In addition, community-based support initiatives should be put in place to provide caregivers with the resources, guidance, and assistance they require, catering to the diverse needs of all caregivers within the community. The key is to ensure that the solutions put in place are comprehensive, accessible, and tailored to the unique needs of each caregiver, empowering them to provide the best possible care for their loved ones by addressing both the educational and community-based aspects.

The observed positive correlation between knowledge, attitude, and practices (KAP) shows the interrelationship of KAP in influencing hypertension care. Caregivers with improved knowledge level demonstrated more positive attitudes and good practices towards hypertension management, indicating the holistic impact of education on caregiver behavior. The results showed an increase in the attitude (from 93.1% to 98.6%) of caregivers towards hypertension with a remarkably good practice (from 78.9% to 85.7%) at week six post-health education, this is in line with a study conducted in Thailand which showed a positive impact after health education was given to caregivers [32]. This also agrees with other studies conducted in sub–Saharan Africa which found that improved knowledge level after health education led to improved positive attitude and good practices [33–35].

The study found that there was good knowledge retention above 90% at week six although the knowledge level score was 2.1% lower than the immediate post-health education score which is the same findings of other studies on knowledge retention [36,37] which found a decrease in the knowledge score after some time suggesting the need for continued health education. Another study on knowledge retention in the Netherlands found no difference in knowledge retention at different testing times after the initial test [38].

The study revealed a negative correlation between age and knowledge retention. This suggests potential challenges in educating older caregivers. Tailored educational strategies may be necessary to effectively address knowledge retention among this demographic group. The older the person gets the less he is going to retain the knowledge gained. This is similar to the study conducted on knowledge retention in denture care which found that the older the participant the less he or she is likely to remember how to care for the dentures [39]. At baseline, there was a fair positive correlation between age and attitude scores, with older participants tending to have more favourable attitude, due to their greater life experience and exposure to hypertension information. However, by week six, attitude became more similar across age groups, because all participants gained comparable knowledge from the intervention, resulting in no correlation between age and attitude. This suggests the need to consider age when using health education as an intervention in disease prevention and management.

The study revealed that, despite having a positive attitude towards managing hypertension, caregivers struggled with practices related to diet and nutrition needs of hypertensive patients at baseline and even at week six assessments. Food insecurity, limited availability of fruits, and reliance on subsistence farming led to diets that are predominantly maize based, lacking diversity, and deficient in essential nutrients [40]. Economic constraints forced households to opt for cheaper, less nutritious foods high in carbohydrates and low in other essential nutrients [41]. Traditional dietary practices and limited access to diverse foods resulted in high salt intake, a well-documented risk factor for hypertension [5]. Post-health educational, caregivers showed improved understanding and practices regarding salt reduction. However, maintaining these changes requires ongoing support and practical solutions that respect local dietary habits and economic realities. Addressing these challenges through comprehensive educational programs, community support initiatives, and policies aimed at enhancing food security and reducing salt intake is essential for improving hypertension outcomes in such resource-limited settings like Malawi.

The findings of this study emphasize the crucial role of health education in enhancing hypertension management, particularly in resource-limited settings like rural Malawi. The significant increase in knowledge level scores immediately post-health education, although with a slight decrease at the six-week mark, suggests the effectiveness of health education in imparting knowledge to caregivers and retaining it. Subsequently resulting in improved positive attitude and good practice. As most of the risk factors for hypertension are modifiable through lifestyle changes, this study gives an insight into how health education can be more useful in enhancing lifestyle changes among rural communities.

### Study strengths and limitations

The study’s strengths include firstly, the utilization of a prospective cross-sectional design, allowing for the examination of knowledge retention and its impact on caregiver behavior over time and secondly, a large sample size (422 caregivers) enrolled in the study, provided a substantial sample size, enhancing the statistical power and reliability of the findings. However, the study has some limitations. The six-week period is shorter to conclude a long-term knowledge retention therefore there is a need to conduct a longitudinal cohort study to assess knowledge retention for a longer period. The study was only conducted in rural areas facing the challenge of generalizability hence the need for a study that will look at both rural and urban areas.

## Conclusion

The baseline assessment revealed that caregivers had limited knowledge level with positive attitude towards hypertension prevention and management and there was a significant gap in their practices, influenced by factors such as education, occupation, age, and gender. Health education had a profound impact on the knowledge, attitudes, and practices (KAP) of the caregivers. It significantly enhanced their knowledge levels, with substantial improvements observed immediately after the education and sustained over a period of six weeks. This education also fostered the maintenance of positive attitude and the adoption of good practices among the caregivers, leading to improved hypertension prevention and management. However, food insecurity limited healthy dietary practices. These findings highlight the crucial role of health education in improving the knowledge level of caregivers of hypertensive patients which can be transformed into positive attitude and good practices in the prevention and management of hypertension. Future interventions should address the unique needs of lower-educated, older, and female caregivers to enhance the effectiveness of hypertension prevention and management strategies in resource-constrained settings.

## Supporting information

S1 File Questionnaire. (DOCX)
