# Supplementary material for: Impact of health education on knowledge retention among caregivers of hypertensive patients: A prospective cross-sectional study in rural Malawi
Source: PLoS One. 2025 Feb 3;20(2):e0317684. doi: 10.1371/journal.pone.0317684 (PMC11790085; doi:10.1371/journal.pone.0317684)
Supplement: S1 File — (DOCX) [file pone.0317684.s001.docx]

**Data Collection Tool**

**Name of Interviewer:** ______________________________  **Date:** _________________

**Identification Number:** ____________**IC3 Clinic:** ⃝ Lisungwi Hospital ⃝ Neno Hospital

**Phone Number:** _____________________________________

**Section 1. Demographic Data**

| **1.1. Age:** | | **1.2. Gender** | ⃝ Male |
| --- | --- | --- | --- |
|  |  |  | ⃝ Female |
|  |  | **1.3. Education Level** | ⃝ No education |
|  |  |  | ⃝ Primary school |
|  |  |  | ⃝ Secondary school |
| **1.4. Relationship with the patient** | ⃝ Spouse |  | ⃝ College graduate |
|  | ⃝ Child |  |  |
|  | ⃝ Parent |  |  |
|  | ⃝ Other (specify) | | |
| **1.5. Location/village:** | | | |
| **1.6. Diagnosis date of Hypertension:** | | | |
| **1.7. Occupation:** | | | |
| **1.8. Religion:** | | | |

**Section 2. Knowledge, Attitudes and Practices**

**2.1. Knowledge of caregivers towards hypertension**

**Note:**

(1) for Correct answer (√ = correct answer for interviewers’ reference)

(0) for Wrong answer

| **ITEM** | **CONTENT** | **YES** | **NO** | **INT**  **REF** |
| --- | --- | --- | --- | --- |
| 1. What is hypertension | A disease that affects the heart and blood vessels |  |  | √ |
|  | High blood sugar |  |  |  |
|  | High blood pressure |  |  | √ |
|  | Affect only older people |  |  |  |
| 1. Risk factors of hypertension | Smoking |  |  | √ |
|  | Age |  |  | √ |
|  | Obesity |  |  | √ |
|  | Sedentary lifestyle |  |  | √ |
|  | Excessive salt intake |  |  | √ |
|  | Excessive alcohol intake |  |  | √ |
|  | Heredity |  |  | √ |
|  | Poor diet |  |  | √ |
| 1. Signs and symptoms of hypertension | Headache |  |  | √ |
|  | vomiting |  |  |  |
|  | Fever |  |  |  |
|  | General body pains |  |  |  |
|  | Heart palpitations |  |  | √ |
|  | Sweating |  |  |  |
|  | General body weakness |  |  |  |
| 1. Complications of hypertension | Heart attack |  |  | √ |
|  | Stroke |  |  | √ |
|  | Kidney damage |  |  | √ |
|  | Heart failure |  |  | √ |
|  | Dementia |  |  |  |
| 1. Prevention of hypertension and its complications | In hypertensive patients, drinking alcohol does not cause complications. |  |  |  |
|  | Minimize salt intake |  |  | √ |
|  | Patients with high blood pressure do not need to quit smoking. |  |  |  |
|  | High levels of stress can lead to a temporary increase in blood pressure. |  |  | √ |
|  | Patients with high blood pressure should not eat fruits and vegetables to increase blood sugar. |  |  |  |
|  | Physical activity can help keep you at a healthy weight and lower your blood pressure. |  |  | √ |
|  | Patients with high blood pressure when suffering from illness such as nausea, vomiting should seek medical attention. |  |  | √ |
| 1. How often should Blood pressure be monitored in hypertensive patient | Daily |  |  | √ |
|  | Weekly |  |  | √ |
|  | Monthly |  |  | √ |
|  | Every 3-6 months |  |  | √ |
|  | Only when the patient is feeling unwell |  |  |  |
|  | No need for monitoring |  |  |  |
| 1. What is a normal BP reading? | 120/80 mmHg |  |  | √ |
|  | 130/80 mmHg |  |  |  |
|  | 140/90 mmHg |  |  |  |
|  | 150/100 mmHg |  |  |  |
|  | Any reading |  |  |  |
| 1. Health education | Have you received any health education or training on hypertension? |  |  | √ yes |
| 1. If yes from the item 8, where did you get the information? | Healthcare provider |  |  | √ |
|  | Internet |  |  | √ |
|  | Support group |  |  | √ |
|  | Community health worker |  |  | √ |
|  | Other (please specify) |  |  | √ |

**2.2. Attitudes of caregivers towards Hypertension**

**Note:**

(3) for Agree; (2) for Not sure; (1) for Disagree

| **ITEM** | **CONTENT** | **Agree** | **Not sure** | **Disagree** |
| --- | --- | --- | --- | --- |
| 1 | Hypertension is a serious condition. |  |  |  |
| 2 | Hypertension can be managed with medication and lifestyle changes. |  |  |  |
| 3 | Caregivers play an important role in managing hypertension. |  |  |  |
| 4 | You are confident in your ability to manage the patient's hypertension. |  |  |  |
| 5 | You are supported by healthcare professionals in managing the patient's hypertension. |  |  |  |
| 6 | You have enough information about hypertension to effectively care for a hypertensive patient. |  |  |  |

**2.3. Practices of caregivers**

**Note:**

(4) for Always; (3) for Sometimes; (2) for Rarely; (1) for Never

| **ITEM** | **CONTENT** | **ALWAYS** | **SOMETIMES** | **RARELY** | **NEVER** |
| --- | --- | --- | --- | --- | --- |
| 1 | Do you ensure that the patient takes their medication as prescribed? |  |  |  |  |
| 2 | Do you encourage the patient to make lifestyle changes such as exercising and eating a healthy diet? |  |  |  |  |
| 3 | Do you monitor the patient's blood pressure regularly? |  |  |  |  |
| 4 | Do you keep a record of the patient's blood pressure readings? |  |  |  |  |
| 5 | Do you communicate with healthcare professionals about the patient's hypertension management? |  |  |  |  |
| 6 | How often do you accompany the patient to doctor's appointments? |  |  |  |  |
| 7 | How often do you prepare meals for the patient that meet dietary guidelines for hypertension? |  |  |  |  |
| 8 | Do you help the patient with meal planning? |  |  |  |  |
